# Anchoring Ethinylestradiol Induced Gene Expression Changes with Testicular Morphology and Reproductive Function in the Medaka

**DOI:** 10.1371/journal.pone.0052479

**Published:** 2012-12-26

**Authors:** Hilary D. Miller, Bryan W. Clark, David E. Hinton, Andrew Whitehead, Stan Martin, Kevin W. Kwok, Seth W. Kullman

**Affiliations:** 1 Integrated Toxicology and Environmental Health Program, Nicholas School of the Environment, Duke University, Durham, North Carolina, United States of America; 2 Environmental Sciences and Policy, Nicholas School of the Environment, Duke University, Durham, North Carolina, United States of America; 3 Department of Environmental Toxicology, University of California Davis, California, United States of America; 4 Department of Statistics, North Carolina State University, Raleigh, North Carolina, United States of America; 5 Department of Environmental and Molecular Toxicology, North Carolina State University, Raleigh, North Carolina, United States of America; Niels Bohr Institute, Denmark

## Abstract

Environmental estrogens are ubiquitous in the environment and can cause detrimental effects on male reproduction. In fish, a multitude of effects from environmental estrogens have been observed including altered courting behavior and fertility, sex reversal, and gonadal histopathology. However, few studies in fish assess the impacts of estrogenic exposure on a physiological endpoint, such as reproduction, as well as the associated morphologic response and underlying global gene expression changes. This study assessed the implications of a 14 day sub-chronic exposure of ethinylestradiol (EE2; 1.0 or 10.0 µg/L EE2) on male medaka fertility, testicular histology and testicular gene expression. The findings demonstrate that a 14 day exposure to EE2 induced impaired male reproductive capacity and time- and dose-dependent alterations in testicular morphology and gene expression. The average fertilization rate/day following the exposure for control, 1.0 and 10.0 µg/L EE2 was 91.3% (±4.4), 62.8% (±8.3) and 28.8% (±5.8), respectively. The testicular morphologic alterations included increased germ cell apoptosis, decreased germinal epithelium and thickening of the interstitium. These changes were highly associated with testicular gene expression changes using a medaka-specific microarray. A pathway analysis of the differentially expressed genes emphasized genes and pathways associated with apoptosis, cell cycle and proliferation, collagen production/extracellular matrix organization, hormone signaling, male reproduction and protein ubiquitination among others. These findings highlight the importance of anchoring global gonadal gene expression changes with morphology and ultimately with tissue/organ function.

## Introduction

Endocrine disrupting chemicals (EDCs) impair reproductive function in diverse wildlife populations [1], [2]. Because the eventual sink for many EDCs is the aquatic medium, fish have been frequently studied. Multiple wild fish populations with altered gonads, in particular testicular oocytes, have been observed around the world including: roach (*Rutilus rutilus*), gudgeon (*Gobio gobio*) and flounder (*Platichthys flesus*) from rivers in the United Kingdom, barbel (*Barbus plebejus*) from Italy, grey mullet (*Mugil cephalus*) in coastal waters of Japan and Korea, shovelnose sturgeon (*Scaphirhynchus platyornchus*) from the Mississippi River and bass species from rivers within the United States [3]–[9]. Many of these gonadal alterations have been attributed to EDC exposures, which are often associated with agricultural practices and municipal effluents. In male fish exposed to estrogenic EDCs, gene expression is altered and reproductive physiology and morphology are subsequently impacted as evidenced by: synthesis of the yolk protein, vitellogenin; altered spermatogenesis; testicular fibrosis; development of testicular oocytes; decreased sperm counts and eventually reduced fertility [7], [10]–[13].

Ethinylestradiol (EE2), the synthetic estrogen in oral contraceptives, is of particular interest from an environmental standpoint due to its high estrogenic potency and its detection in aquatic systems receiving sewage treatment water [14]–[18]. A 7-year study in northwestern Ontario, Canada in which an entire lake was regularly dosed with environmentally relevant concentrations of EE2 (4.8–6.1 ng/L) for three years demonstrated impaired spermatogenesis and testicular oocytes in both pearl dace (*Margariscus margarita*) and fathead minnow (*Pimephales promelas*) populations [19], [20]. Furthermore, there was a collapse of the fathead minnow population after the second year due to loss of young-of-the-year individuals [19].

Studies on wild roach (*Rutilus rutilus*) downstream of wastewater treatment facilities in the United Kingdom found vitellogenin induction and testicular oocytes in males [7]. Subsequent laboratory studies of roach to environmentally relevant EE2 concentrations found strong effects on male gonadal development and reproduction [21], [22]. Laboratory studies of model fish, such as zebrafish (*Danio rerio*) and medaka (*Oryzias latipes*), have also demonstrated decreased reproductive capacity following EE2 exposure [23], [24].

The endocrine system is a complex, integrated system with the ability to influence expression of a wide range of genes. Proper steroid hormone signaling and reproduction function is dependent on the hypothalamus-pituitary-gonadal (HPG) axis. EDCs can influence multiple points within the HPG axis [25]. Surprisingly, few studies have assessed global gonadal gene expression changes associated with EE2 exposure in fish. The majority of studies, for example, have targeted specific genes involved in steroid hormone biosynthesis or known estrogen-responsive genes [11], [12], [23], [26]. Hirakawa et al. [27] assessed testicular gene expression in EE2 exposed adult medaka with a strong emphasis on genes associated with testicular oocytes. Since testicular oocytes are one of the traits often used as indicators of chronic estrogen exposure in fish, this study identified associated gene expression profiles of testicular oocytes as potential biomarkers. In this study, we focused on sub-chronic testicular gene expression events and associated morphological changes that may occur prior to formation of ovo-testis including increased germ cell apoptosis, decreased germinal epithelium and thickening of the interstitium.

The aim of this study was to further understand the molecular mechanisms of estrogen induced toxicity by linking testicular gene expression pathways with altered testicular morphology and reproductive function. To achieve this aim, we used a sub-chronic 14 day exposure to EE2 as our treatment regimen and followed with three assessments: (1) a breeding study to assess the reproductive effect of a 14 day EE2 exposure on the male fish; (2) a histological examination of the testis on days 1, 7 and 14 of the EE2 exposure and following a post exposure 14 day depuration period; and (3) testicular gene expression analysis through a medaka specific microarray on days 1, 7 and day 14 of the EE2 exposure.

## Results

### Reproductive Assessment

**Figure 1:**
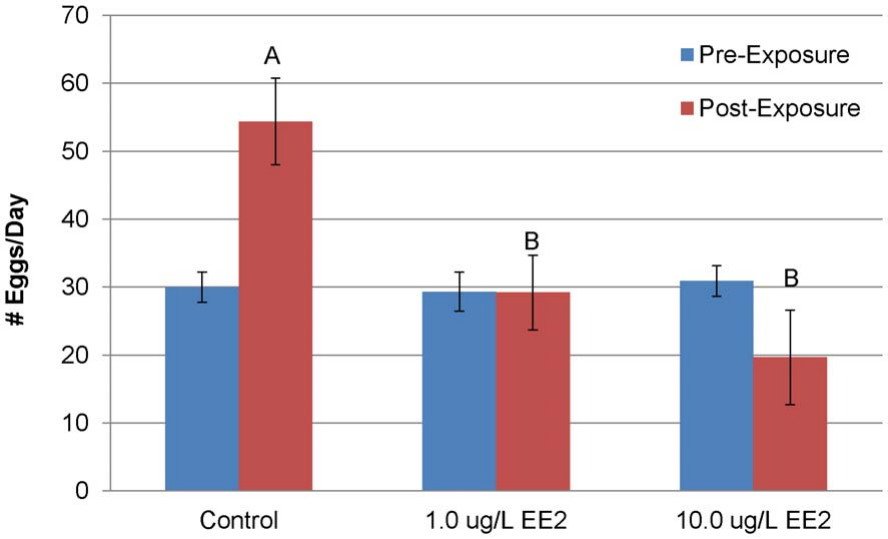
Number of eggs produced per day for medaka breeding groups during the pre- and post-exposure period following a 14 day exposure to EE2 period (mean ± SEM). No statistical difference was found during the pre-exposure period. One-way ANOVA of the post-exposure time period found significant differences between the control and treatment groups. Different letters indicate statistical difference in the post-exposure time period (p<0.05).

**Figure 2:**
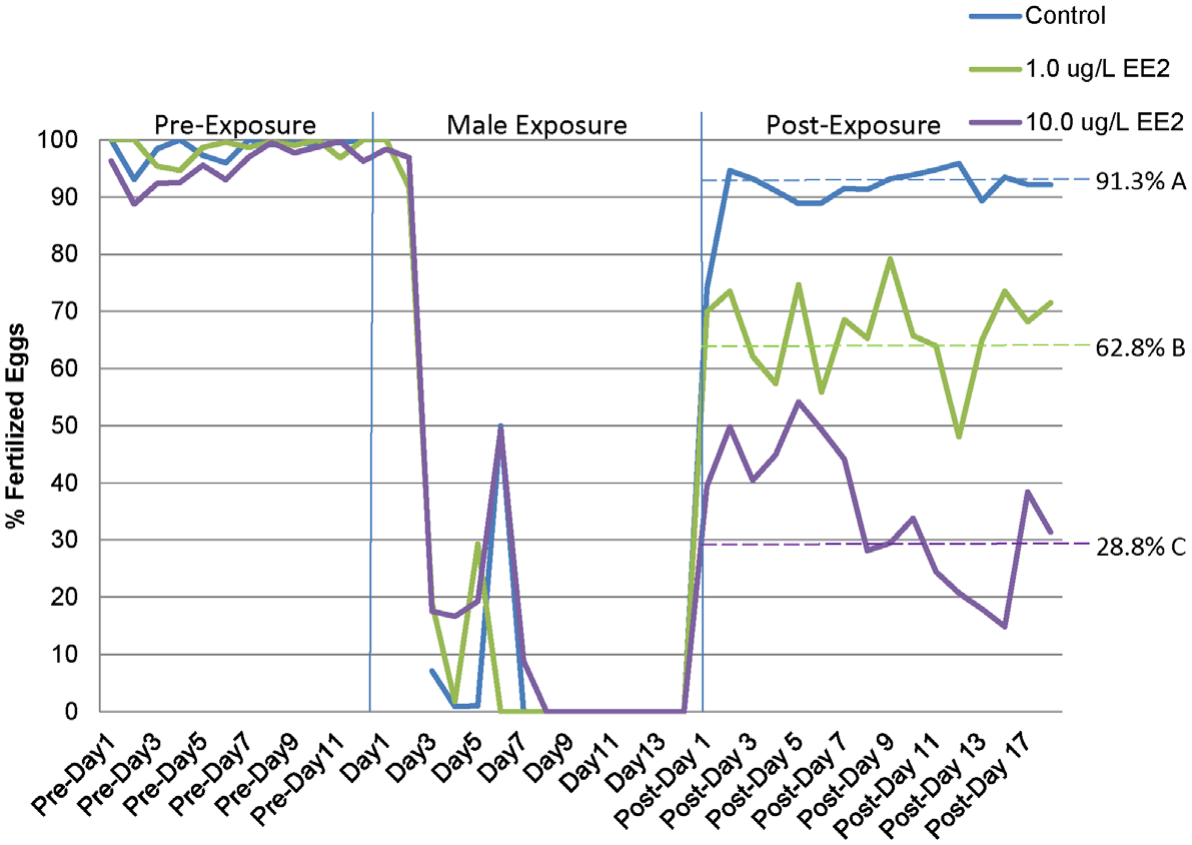
Percentage of eggs fertilized for medaka breeding groups during three periods: pre-exposure, male exposure, and post-exposure. Dashed lines indicate the mean percentage fertilized during the 20-day post-exposure period for each treatment. Different letters indicate a statistical difference (p<0.05).

During the 12 day pre-exposure period, breeding groups consisting of one male and three females were established and monitored daily for fecundity and fertility. All groups were reproductively active with no statistically significant difference in daily fecundity or fertility (p>0.05) (Figures 1 and 2). Upon removal of males from the breeding group (for male-only exposure to EE2), residual egg production in females was sporadic and greatly reduced. Fertilization of eggs ceased 8 days following removal of males indicating residual sperm were retained in the breeding chambers following removal of males (Figure 2).

Males were exposed to DMSO (control), 1.0 µg/L EE2 or 10.0 µg/L EE2 for 14 days. Following the male exposure and the designed return of males to their respective breeding chambers, there was a significant decrease in fecundity (the total number of eggs/day) in both EE2 treatments. While control beakers averaged 54.4 (±6.4) eggs/day (n = 6), the 1.0 (n = 6) and 10.0 (n = 7) µg/L EE2 beakers averaged 29.2 (±6.9) and 19.7 (±3.8) total eggs/day respectively (Figure 1). Both the 1.0 and 10.0 µg/L EE2 treatments were significantly different from control (p<0.05) but not from each other (p = 0.16). The fertilization rate of eggs laid/day exhibited a significant concentration response. The average percent of eggs fertilized/day for control, 1.0 and 10.0 µg/L EE2 was 91.3% (±4.4), 62.8% (±8.3) and 28.8% (±5.8), respectively (Figure 2). The 1.0 µg/L EE2 treatment was significantly different from control and the 10.0 µg/L EE2 treatment was significantly different from both control and 1.0 µg/L EE2 treatment. At the end of the post-exposure breeding period fertilization rates and fecundity remained at decreased levels suggesting recovery of reproductive capacity was not achieved during the 20 day time frame of our post exposure reproductive study.

### Histology

The following cohorts of fish were assessed histologically: control non-breeding males; exposed non-breeding males on days 1, 7 and 14 of EE2 exposure; exposed non-breeding males allowed to recover for 14 days post exposure; control breeding males and exposed breeding males from the reproduction experiment.

#### Control males

**Figure 3:**
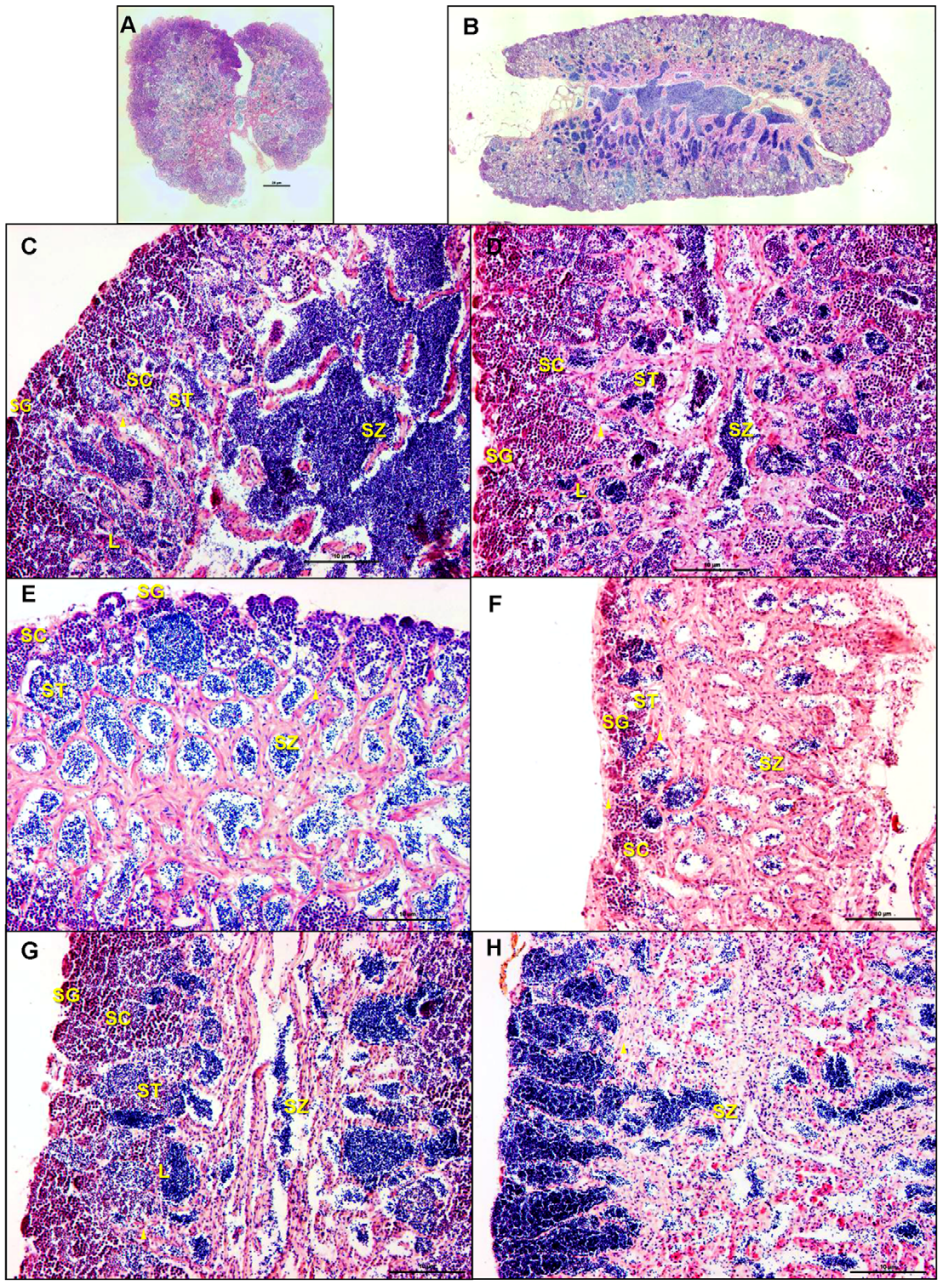
Sections of testis from 6 month old male medaka stained with H&E. A: Transverse section of testis showing both lobes joined by the central efferent duct. Spermatogonial germ cells are at periphery of the organ. B: A longitudinal section of a DMSO control animal showing normal testicular morphology. Dark basophilic staining central region is comprised of spermatozoa filling lumen of efferent duct. As in A (above) spermatogonial cells are restricted to the periphery of the organ. Between the periphery and the CED various stages of spermatogenesis are encountered. C: Animal exposed to 1.0 µg/L EE2 for 7 days. When interstitium of animals from this group were compared to controls only one animal showed enhanced thickening of the interstitium and this was limited in extent. D: Animal exposed to 10.0 µg/L EE2 for 7 days. Thickened interstitium was apparent in a zone half way between the periphery and the CED. E: Animal exposed to 1.0 µg/L EE2 for 14 days. At this time, moderate thickening of the interstitium and increased area of clear space devoid of germ cells characterized the lobular lumen. There is a decrease in the proportion of intermediate staged germ cells as evident by the smaller area occupied by these cells (i.e. spermatocytes and spermatids) and a general decrease in germinal epithelium. F: Animal exposed to 10.0 µg/L EE2 for 14 days has a severe thickening of the interstitium, increased vacuolization, and an overall decrease in germinal epithelium. G: Animal exposed to 1.0 µg/L EE2 for 14 days followed by 14 days of recovery showed return toward control morphology. The thickening of the interstitium has diminished compared to E or F and there is an abundance of intermediate stage germ cells indicating active spermatogenesis. The CED does not have many spermatozoa but there are spermatids and spermatozoa in the efferent duct system preparing to enter the central duct. H: Animal exposed to 10.0 µg/L EE2 for 14 days followed by 14 days of recovery. There is continued thickening of the interstitium and significantly altered spermatogenesis. Only mature sperm and a few spermatogonia are present but intermediate stages of germ cells are absent or greatly reduced indicating little active spermatogenesis. There is also eosinophilic change in the interstitium.

Histological assessment of control medaka (Figure 3A and B) demonstrates that the testis is bilobed with a restricted spermatogonial lobular structure composed of lobules that end blindly at the periphery of the organ. This is similar to testicular organization of medaka reported in the literature [28]–[31]. In fish with this testicular architecture, spermatogonia are only present at the periphery of the lobule. Based on our results and those described by others, Sertoli cells surround individual spermatogonia forming an isogenic cyst that undergoes synchronized development through spermatogenesis [31]–[34]. Further analysis revealed that the testis is divided into two compartments: the lobular compartment, consisting of germ cells and Sertoli cells, and an interstitial compartment, comprised of Leydig cells, peritubular myoid cells, connective tissue, efferent duct epithelial cells, endothelium of blood vessels and circulating blood cells [29], [31], [33]–[36].

Testicular interstitium stained with a pronounced eosinophilia while germinal epithelium showed more prominent basophilic staining. As is shown in Figure 3A and B, the mature spermatozoa showed greatest amount of basophilia seen particularly well in the central efferent duct. Lumens of the lobules were nearly completely filled with rounded cells whose morphology was consistent with germinal epithelium. As the lobular lumens were followed centrally, they were occupied by cells whose nuclei stained extremely basophilic (i.e., dark purple). Curvy elongated eosinophilic structures marked the interstitium. This material provided sufficient resolution to identify specific compartments and cell types and spermatogenic stages in the testis.

#### Exposed non-breeding males

Animals were exposed to DMSO (control), 1.0 µg/L EE2, and 10.0 µg/L EE2 for 14 days and sampled for histology on exposure days 1, 7 and 14. Analysis demonstrated both time- and concentration-dependent alterations in testicular structure including: thickening of the interstitial tissue, increased apoptosis of germ cells, and altered spermatogenesis as evidenced by decreased proportion of spermatocytes and spermatids in germinal epithelium (Figure 3C–F). Apoptotic germ cells were identified by their shrinkage, nuclear condensation, and fragmentation into spherical, membrane-bound bodies. The latter are often phagocytized by neighboring cells as previously described [37]. No change in testicular morphology was observed on day 1 of exposure. On exposure day 7, one individual from 1.0 µg/L EE2 treatment (n = 3) exhibited thickening of the interstitial tissue and decreased spermatozoa. The 10.0 µg/L EE2 treatment (n = 3) after 7 days of exposure exhibited a similar thickening of the interstitial tissue as well as increased apoptosis of spermatocytes and spermatids (Figure 3D). On day 14 of exposure, all individuals in the 1.0 µg/L EE2 treatment (n = 3) exhibited thickening of interstitial tissue and increased apoptotic spermatocytes and spermatids. In addition, one replicate also displayed a decrease in spermatozoa (Figure 3E). The 10.0 µg/L EE2 treatment (n = 3) on exposure day 14 displayed increased thickening of the interstitial tissue as well as altered spermatogenesis and a loss of germinal epithelium (Figure 3F).

#### Recovery of exposed non-breeding males

Following the 14 day EE2 exposure, male fish were allowed to recover for an additional 14 day depuration (recovery) period. Individuals exposed to 1.0 µg/L EE2 (n = 3) exhibited signs of recovery following the depuration period (Figure 3G). Two of these replicates had an increased proportion of spermatocytes with concomitant decreased proportion of spermatozoa, indicative of initial resumption of spermatogenesis. However, the thickened interstitium persisted. The third replicate showed presence of testicular oocytes, thickened interstitium and many spermatozoa. Following 14 day depuration, individuals exposed to 10.0 µg/L EE2 exhibited continued thickening of interstitial tissue, eosinophilic change in the interstitium (likely proteinaceous fluid), altered spermatogenesis, and a decrease in germinal epithelium (Figure 3H). Testicular oocytes were observed in one of the 10.0 µg/L EE2 replicates.

#### Exposed breeding males from the reproductive experiment

**Figure 4:**
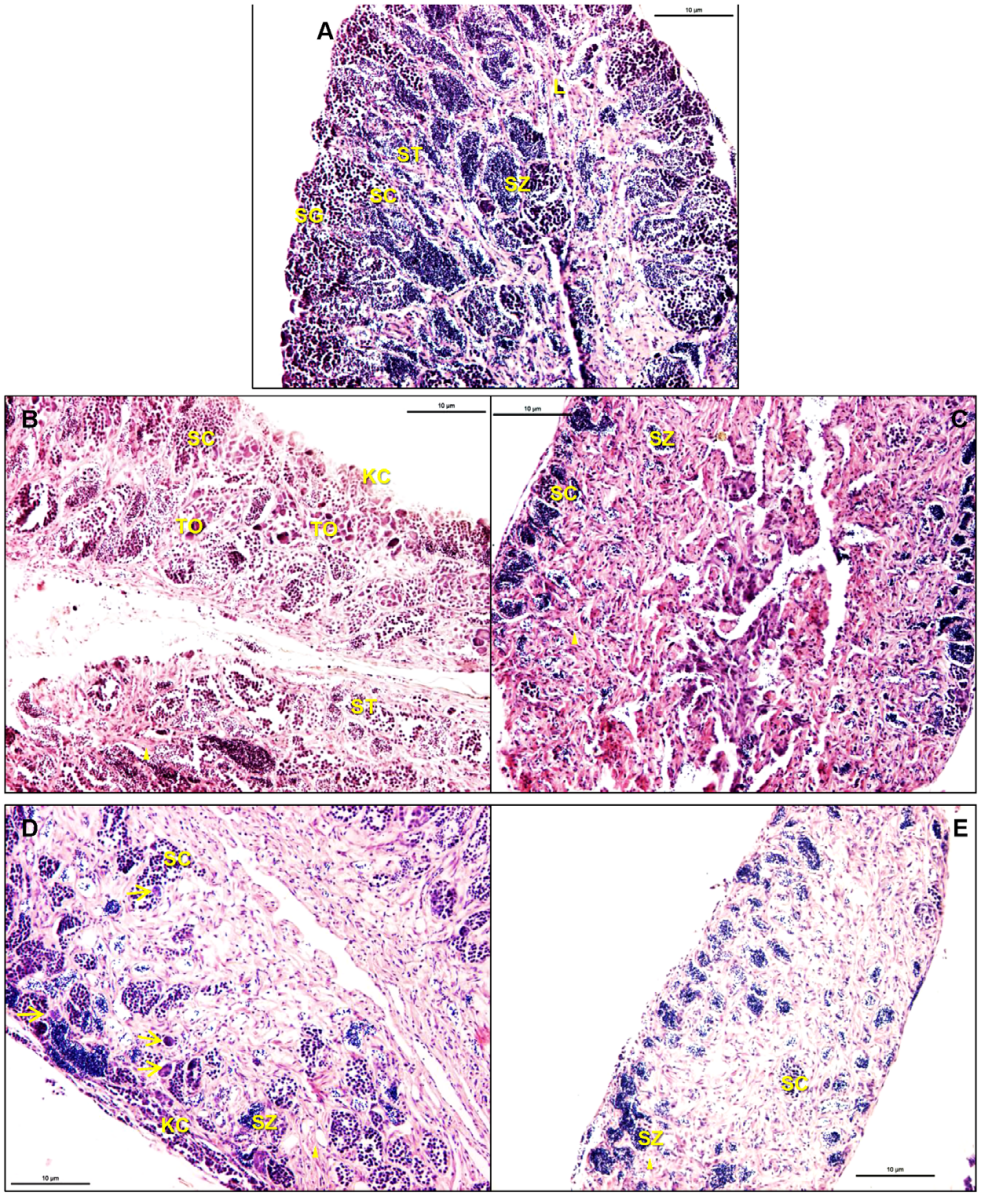
Testis of medaka following a 14 day EE2 exposure followed by active breeding with 3 females for 20 days. A: DMSO control male. B: Male exposed to 1.0 µg/L EE2. There is thickening of the interstitium, an increase in proportion of spermatocytes, minimal spermatozoa. C: Male exposed to 1.0 µg/L EE2. This organ has severe thickening of the interstitium with a focal area of basophilic cells center of field and a severe decrease in germinal epithelium. D: Male exposed to 10.0 µg/L EE2. Severe thickening of interstitium and severe loss of germinal epithelium are. There are, however, spermatocytes and spermatids present suggesting active spermatogenesis. E: Male exposed to 10.0 µg/L EE2. There is severe thickening of the interstitium with loss of germinal epithelium.

An assessment of the testicular morphology of EE2 treated breeding males following the reproductive assessment demonstrated alterations similar to those of testes from the non-breeding males on day 14 of exposure. In some instances, however, there was further degeneration of the testis of the breeding males versus the testis of non-breeding males immediately following the 14 day EE2 exposure. The testis from a single control breeding male contained testicular oocytes, karyomegalic germ cells, and disorganized lobular space. While spermatogenesis was occurring in this animal (i.e., spermatozoa were present), the altered morphology and general disorganization of the lobular space was significant. All other controls appeared normal. Male fish exposed to the 1.0 µg/L EE2 treatment (14 days, n = 4) followed by 20 days of breeding had altered germ cell epithelium and mild to severe thickening of the interstitium compared to controls (n = 5). This ranged from an increase in intermediate stage germ cells (spermatocytes and spermatids) and a decrease in spermatozoa associated with mild to moderate thickening of the interstitium to a severe loss of germ cell epithelium which was associated with maximal thickening of the interstitium (Figures 4B&C). Testicular oocytes and karyomegalic germ cells were found in three of the four replicates. The gonad without testicular oocytes had severe depletion of the germ cell epithelium and severe thickening of the interstitium. Male fish exposed to the 10.0 µg/L EE2 treatment (14 days, n = 6) followed by 20 days of breeding exhibited more pronounced alterations than did those of the 1.0 µg/L EE2 treated males. All animals had a moderate to severe thickening of the interstitium, which was also associated with a significant loss of germ cell epithelium (Figures 4D&E). Testicular oocytes and karyomegalic germ cells were present in four of the six gonads assessed. As seen in the 1.0 µg/L EE2 treated breeding males, gonads without testicular oocytes had severe thickening of the interstitium and significant loss of germ cell epithelium.

### Gene Expression

**Figure 5:**
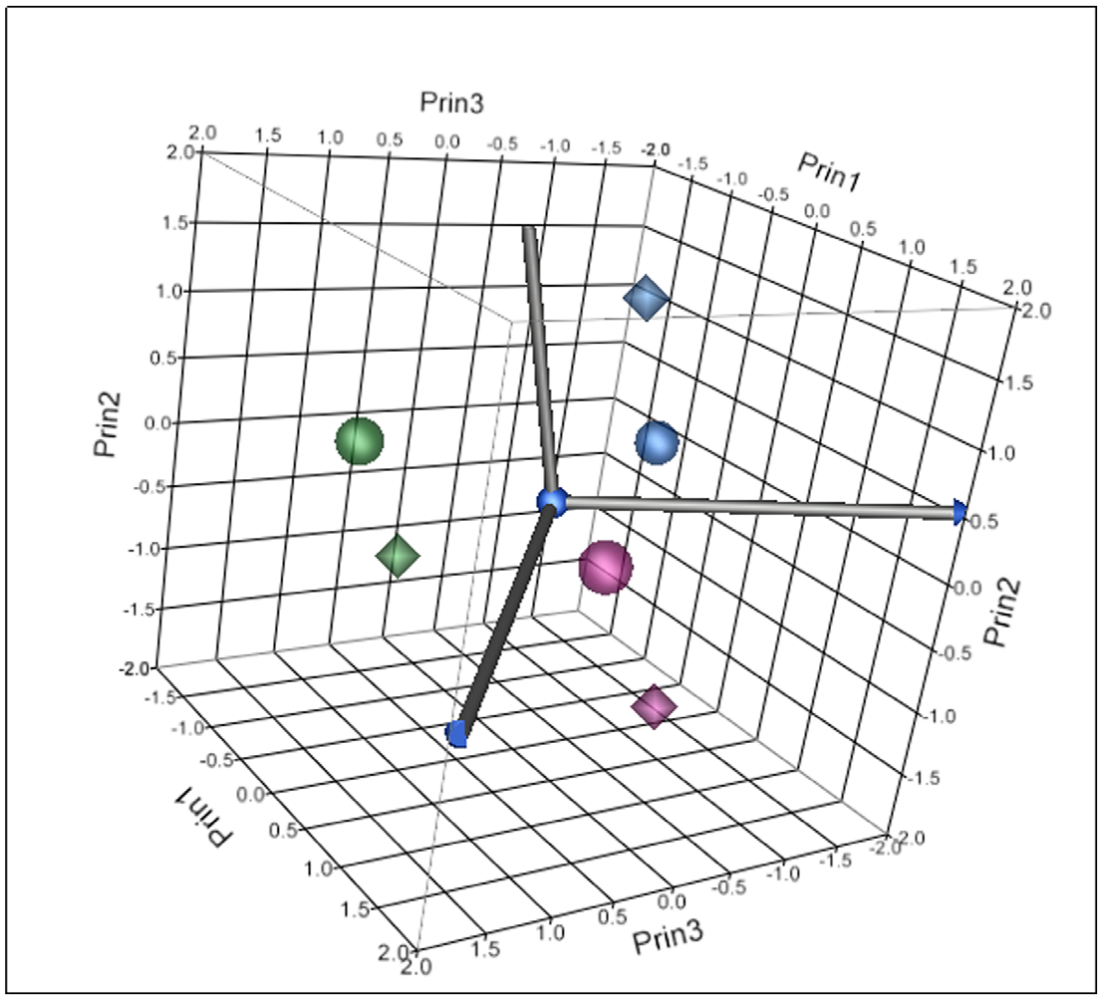
Principle components analysis based on significant genes from 1.0 µg/L EE2 treatment group (circles) and 10.0 µg/L EE2 treatment group (diamond) on day 1 (purple), day 7 (blue), and day 14 (green). The first principal component accounted for 47.7% of the data variability and was largely influenced by the strong temporal response in each EE2 treatment. The second (16.3%) and third (13.8%) principal components appeared to be influenced by dose accounting for a combined 77.8% of the total data variability.

**Figure 6:**
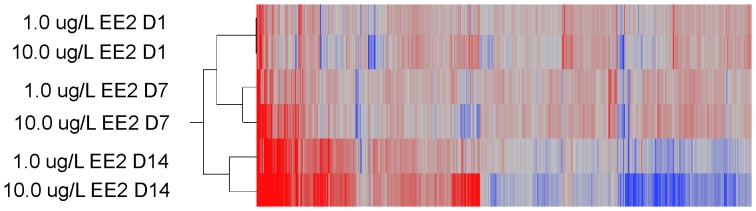
Hierarchical cluster diagram of significantly different genes following exposure to EE2 in males. A temporal trend is clearly observed. The same day sampling points cluster together with day 14 being the most distinct from day 1 and 7.

Testes were sampled on days 1, 7 and 14 of exposure for gene expression analysis. Significant differences in gene expression were observed between control and treatment groups at all sampling time-points. Analysis of gene expression data for 1.0 µg/L EE2 and 10.0 µg/L EE2 treatments on days 1, 7, and 14 of exposure demonstrated time- and concentration-dependent alterations. Time and concentration response trends were evident in both principle components analysis (PCA) (Figure 5) and hierarchical clustering (Figure 6). Assessment of principal components demonstrated a clear temporal trend as well as a concentration-dependent trend (Figure 5). The first principal component accounted for 47.7% of the data variability and was largely influenced by the strong temporal response in each EE2 treatment. After considering the temporal contribution to the variation in gene expression data, the second (16.3%) and third (13.8%) principal components appeared to be influenced by concentration accounting for a combined 77.8% of the total data variability. Evident in both PCA and hierarchical clustering, gene expression alterations on days 1 and 7 appeared more similar than gene expression changes on day 14.

**Figure 7:**
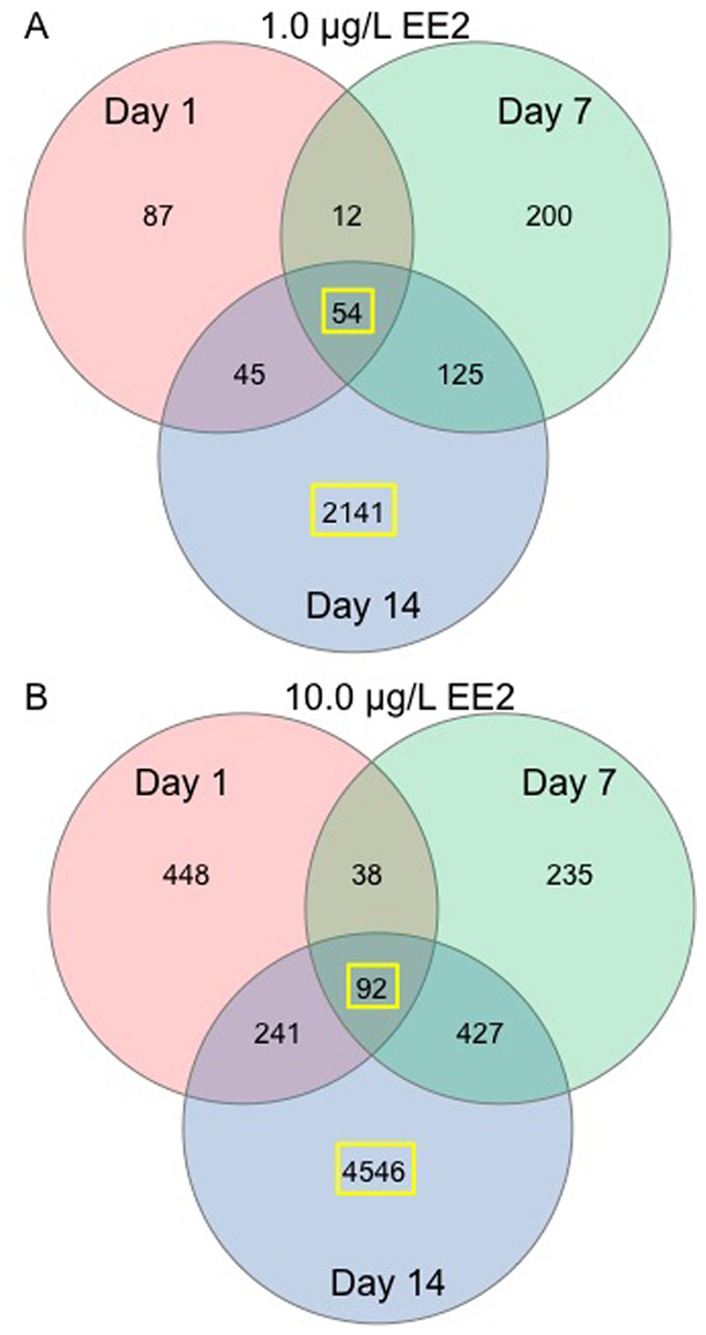
Venn diagrams of significantly different genes on day 1, day 7 and day 14 of EE2 exposure for a) the 1.0 µg/L EE2 treatment group and b) the 10.0 µg/L EE2 treatment group. The 54 common genes in the 1.0 µg/L EE2 treatment group, the 92 common genes in the 10.0 µg/L EE2 treatment group, the 2141 genes unique to day 14 1.0 µg/L EE2 treatment group, and the 4546 unique genes to day 14 10.0 µg/L EE2 treatment group, highlighted by the yellow box, were used for Ingenuity Pathway Analysis.

Examination of data on a gene by gene basis relative to same day control demonstrates both a time- and concentration-dependence regarding the number of significant genes with the least number of significant transcripts expressed in the 1.0 µg/L EE2 treatment on day 1 and the most in the 10.0 µg/L EE2 treatment on day 14. On day 1 of exposure there were 198 and 819 significant transcripts differentially expressed in 1.0 µg/L EE2 and 10.0 µg/L EE2 treatments respectively (Figure 7A and B). Of these responses 87 of the 198 transcripts were unique in the 1.0 µg/L EE2 treatment and 448 of the 819 transcripts were unique in the 10.0 µg/L treatment. Similar patterns were observed for days 7 and 14 with 391 total transcripts in the 1.0 µg/L EE2 treatment on day 7 (200 unique), and 2365 transcripts in 1.0 µg/L EE2 treatment on day 14 (2141 unique) (Figure 7A). The 10.0 µg/L EE2 treatment on day 7 had 792 total transcripts (235 unique) and day 14 had 5306 total transcripts (4546 unique) (Figure 7B). Of the significantly expressed transcripts, there were 54 genes common to all sampling days (1, 7 and 14) in the 1.0 µg/L EE2 treatment (Figure 7A) and 92 common genes expressed in the 10.0 µg/L treatment (Figure 7B). Finally, there were 33 differentially expressed genes common to all treatments and time-points (Table 1). Gene ontology of these genes indicates predominant involvement in cellular structure and extracellular matrix, cell proliferation, apoptosis, transcription, hormone signaling and male reproduction. Additionally, multiple nuclear receptors involved in reproductive hormone signaling were differentially expressed during all doses and time-points including NR0B1 (DAX1), NR0B2 (SHP1), NR3C3 (PGR) and NR5A1 (SF-1).

**Table 1:** Significant genes common to all sampling points and treatments.

Significant Genes Common ToAll Treatments:	ACTA1, ACTG1, ADAMTS20, BMP6, CALD1, CD81, CDH5,COL4A1, COL4A2, CXXC5, CYP19A1, EFEMP2, EPB41, FAM187A, FAM20C, FLNC, FOLR1, GPC1, ID1, KCNS2, MYOCD, NR0B1, NR0B2, NR3C3, NR5A1, PDLIM3, PGM5, PTPRN, SLPI, SOX9, TGM2, TULP1, VENTX, UNKNOWN (ENSORLG00000006007, ENSORLG00000009404)

Ingenuity Pathway Analysis (IPA) was employed to assess the signaling pathways and functionality of gene expression changes using gene lists representing the following differentially expressed transcripts (as highlighted in Figure 7A and B): 1) genes common to all sampling days in the 1.0 µg/L treatment group (54 genes); 2) genes common to all sampling days in the 10.0 µg/L treatment group (92 genes); 3) genes unique to day 14 of exposure in the 1.0 µg/L treatment group (2141 genes); and 4) genes unique to day 14 of exposure in the 10.0 µg/L treatment group (4546 genes). The top Molecular Networks and their associated genes from the IPA analysis within each of the above differentially expressed gene sets are listed in Tables 2 and 3. These networks had scores between 16 and 64 indicating likely interactions between each network and focus genes from the input data set. Each network was assembled with 35 focus genes permitted. Canonical Pathways generated from differentially expressed genes unique to day 14 for both the 1.0 µg/L and 10.0 µg/L EE2 treatments are listed in Table 4. These pathways reflect many of the genes and molecules highlighted in the Molecular Networks. A further comparison of the Molecular Networks generated from gene lists of the differentially expressed genes common to all days in the 1.0 µg/L and 10.0 µg/L EE2 treatments indicated multiple network molecules and genes identified in both treatments. Gene ontology of these network genes indicates involvement in multiple biological functions including apoptosis and cell death, cell cycle and proliferation, collagen, cytoskeletal organization, hormone signaling, male gonad function and others (Table 2). Of these genes and molecules, multiple examples were seen with important and well known involvement in hormone signaling and male reproduction including CYP19A1, FSH, GPER, Insulin, Lh, NR0B1, NR0B2, NR5A1, Smad, and SOX9. Gene expression and pathway analysis also suggests a perturbation of important mitogenic and apoptotic signaling pathways throughout the EE2 exposure, including ERK1/2, NFκB, P38, MAPK, PDGF, PKA, Smad, and TGF beta. Interestingly, an increased prevalence of differentially expressed genes within these pathways was associated with increased exposure concentration and time.

**Table 2:** IPA top associated molecular networks function generated from gene lists comprised of significant genes common to all sampling days in each treatment.

Significant Gene List	Top Associated Molecular Network Functions	Network Genes and Molecules	Ontology of Genes and Molecules Common to 1.0 and 10.0 IPA Networks
1.0 µg/L EE2 common to all sampling days	1. Embryonic Development, Organismal Development, Organ Development (Score = 46)	**ACTA1, ACTG1, Actin, Alpha actin, BMP6, CALD1, collagen, COL4A1, CYP19A1, EPB41, F ACTIN,**Fibrin, **FLNC,** FSH, **ID1,** Insulin, Histone h3**,** **Histone h4,** Lh, **MYOCD,** NFκB (complex)**,** **NR0B1, NR0B2, NR5A1,** **PDLIM3,** Pka, **PTPRN,** **TAGLN,** Tgf beta, **TGM2,** PDGF BB, **SLPI, Smad,** **SOX9, TPM1, TROPOMYOSIN**	**angiogenesis and wound healing:** EFEMP2, Fibrin, FYN, Histone H3, ID1, P38 MAPK, PDGF BB, Pka, Smad, TGF beta**apoptosis and cell death:** CXXC5, ID1, NFκB complex, P38 MAPK, TGF beta, TGM2, TULP1**cell cycle and proliferation:** BMP6, CCND1, CD81, ELK1, ERK1/2, MYOCD, P38 MAPK, PDGF BB, Pka, Smad, SOX9, TGF beta
	2. Connective TissueDisorders, Genetic Disorder, Dermatological Diseasesand Conditions(Score = 23)	BRS3, CAV1, **CD81**, **CDH5**, **COL12A1**, COL13A1, COL20A1, COL21A, COL22A1, COL23A1, COL27A1, COL28A1, **COL4A2**, COL6A5, COL6A6, COL8A2,collagen, **CXXC5**, EGFR, **EMID1**, FN1, **FOLR1**, **GJB2**,GPR146, GPR160, HNF4A, IFNG, Mmp, PEPD,**PNPO**, **PTGER1**, **SMAD9**, TTC37, VN1R1,ZNF557	**collagen:** COL4A1, COL4A2, TGF beta**cytoskeletal organization:** ACTA1, ACTG1, Actin, Alpha actin, CALD1, EPB41, F actin, FLNC, PDGF BB, PDLIM3**hormone signaling:** CYP19A1, FSH, Insulin, Lh, NR0B1, NR0B2, NR5A1
	3. Lipid Metabolism, Liver Cholestasis, Molecular Transport (Score = 16)	**C1QL1**, C1QTNF2, C2ORF40, **CADM4**, CCND1, CCND3, Dcc dimer, DNER, **EFEMP2**, ERBB3, ERK1/2, ETS-ELK1, FGF18, FGFR1, FOS, FYN, GAS5, GLP2R, **GPC1**, HTR6, IL17RD, IL22R1-IL10R2, KLB, MSK ½, NR3C1, P38 MAPK, PAX3, PCDHGC3, SLFN1, SRF-ELK1, **ST8SIA2**, **SVIL**, **TULP1**, **VENTX**, ZNF335	**inflammatory response:** BMP6, CXXC5, NFκB complex, P38 MAPK, Smad, TGF beta**male gonad function:** BMP6, CCND1, FSH, NR0B1, NR0B2, NR5A1, Smad, SOX9**response to stimulus:** FYN, GPC1, P38 MAPK, TULP1
10.0 µg/L EE2 common to all sampling days	1. Cancer, ReproductiveSystem Disease,Drug Metabolism(Score = 64)	**ACTA1, ACTA2, ACTG1, Actin, Alpha actin, BMP6, CALD1, CXXC5, CYP17A1, CYP19A1, DTNA,** **EPB41, F Actin, FLNC,** FSH, hCG, **ID1, INHA,** Lh,**MCAM, MYOCD, NR0B2, NR0B1, NR5A1,** **NR5A2,** NFκB (complex)**, PDLIM3,** **PNPT1, PTPRN, RGS4, RNF7,** RNA polymerase II,**SLP1, Smad, SMAD7**	**telomere maintenance:** Histone H3, Histone H4**transcription:** ID1, NFκB complex, Smad, VENTX**undetermined:** SLP1
	2. Cell Cycle, Cellular Development, Hematological System Development and Function (Score = 30)	2810007J24RIK, beta-estradiol, CCND1, **CXCL14**, **DDAH1**, DSEL, **EFEMP2**, FGF2, **FOLR2**, FYN, **GAB3**, **GPC1**, **Gpcr**, GPR174, **GPR182**, GPR89A, GPR89C, **HOXA3** (includes EG:3200), **HS3ST2**, **HS3ST3A1**, HS3ST4, **NBEA** (includes EG:26960), NDST4, OPN1MW2, PPARA, retinoic acid, **RIN3**, **SC5DL**, SRC, **sulfotransferase**, thyroid hormone, TP53, **TULP1**, Ubiquitin, **VENTX**	
	3. Embryonic Development, Tissue Morphology, Cardiovascular System Development and Function (Score = 27)	ADCY, Akt, Alp, Ap1, **CD81,** **CD151,** **CDH5,** **COL4A1,** **COL4A2,** **Collagen type IV,** Creb, ERK1/2, **FBLN5,** Fibrin, **FSHR,** **GDF6,** **Gpcr,** Histone h3, Histone h4, **HSF1,** IL1, Insulin, LDL, **LPL,** Mapk, P38 MAPK, PDGF BB, Pka, PI3K complex, **RXFP4,** **SORL1,** **SOX9,** Tgf beta, **TGM2,** Vegf	

Significantly different genes listed in bold.

**Table 3:** IPA top associated molecular networks function generated from gene lists comprised of significant genes unique to day 14 in each treatment. Significantly different genes listed in bold.

Significant Gene List	Top Associated Network Functions	Network Genes and Molecules	Ontology of Genes and Molecules Common to 1.0 and 10.0 IPA Networks
1.0 µg/L EE2 genes unique to Day 14	1. Post-Translational Modification, Protein Degradation, ProteinSynthesis (Score = 40)	**AHNAK, APBB2, ARIH1, CDC34, c-Src, EGFR, GOT1, GPM6B, GRLF1, MGRN1, MXI1, NDFIP2, PLRG1, PRCC, RNF13, RNF25, RNF146, RNF167, RNF181, RNF185, RUSC1, SCAMP3, SLC11A2, SLC22A18, SPARCL1, SPDEF, SPG20, SOX17, UBE2, UBE2D1, UBE2H, UBE2J2, UBE2L3, UBE2N, UBE2V2**	**glycolysis:** DLAT, PDHA1, PDK2, PDK4**transcription and angiogenesis:** RBM15**translation:** EEF1B2
	2. Lipid Metabolism, Nucleic Acid Metabolism, Small Molecule Biochemistry (Score = 37)	**ABCC9,** Aconitase, **ACTN1, C12ORF44, CSTF2, DLAT, EEF1B2, EEF1G,** ERK1/2, **FXN, JDP2, KCND2, KSR1, LONP1, MGAT3, MICALL1, MOBKL1B, P13A1, PDHA1, PDK2, PDK4, PLEK, RALGPS2, RBM15, SAMD4B, SLC12A4, STK38L, SURF2, VARS,** VLDL, **VRK2, WDR20, UCN3, USP46, YWHAB**	**ubiquitination:** DUB, USP30, USP37, USP46, WDR20**undetermined:** SURF2
	3. Post-Translational Modification, GeneExpression, MetabolicDisease (Score = 35)	**ARNT, ARNT2, BAG4, BST1, CALCOCO1, CDC371, CDKN2D, CRKL, DNAJC13, DNAJC16, DUB, ERBB2, Estrogen Receptor, HSD17B12, Hsp22/Hsp40/Hsp90,** Hsp90, **NKX6-2, P4HA2, PQLC1, SCG5, SELENBP1, SQLE, TBC1D17, TMEM43, UCHL1, USP4, USP7, USP30, USP37, USP45, USP47, WSB2, ZDHHC14, ZFHX3, ZFP36L1**	
	4. Developmental Disorder, Genetic Disorder,Neurological Disease(Score = 33)	**ACADL, ACY1, APPBP2, BBS2, BBS4, BBS7, BCAT2, BCKDHB, BCL11A, CHMP5, CORO1A, EPHX2, FSTL1, G0S2, Gpd, GPD1, ITPKB, lymphotoxin-alpha1-beta2, MUL1, NACA,** NFκB (complex)**, PAK1IP1, PASK,** peptidase, PPARα-RXRα, **PRDX2, PRDX4, SCFD1, SLC2A5, SLC27A1, TNFAIP2, TRAFD1, TRPC4AP, UBA52, WDR34**	
	5. Protein Degradation,Protein Synthesis,Embryonic Development(Score = 33)	**AMFR, CUL1, DAF7, DERL1, DERL2,** Elastase, **ELF4, ETS, FAF1, FAF2, FBXL20, FBXO4, FBXO7, FBXO31, FBXO33, FLT1, HERC3, KLHL22, KLHL13, NRP1, NSFL1C,** P38 MAPK, **PCGF2, PHC2, RAB4B, RAB7A, RABGGTB, RNF126, Scf Trcp beta, SFMBT1, TNNI1, UBE2T,** Ubiquitin, **UFD1L, VPS36**	
10.0 µg/L EE2 genes unique to Day 14	1. Cell Cycle, ConnectiveTissue Developmentand Function, CarbohydrateMetabolism (Score = 34)	**ANLN, CDR2, CEBPZ, CEP57, CHD5, CTPS, DDX18, DIAPH3, FANC1, GAMT, HEATR1, KLHL5, METTL13, MRPL12, MUM1, MXD4, MYC, MYCN, MYCT1, NCOA5, NOSIP, OMG, PLA1A, RPL5, RFX3, RPL13, RPS12, RPS23, SCAMP1, SCPEP1, SLC25A19, TPP2, WAC, XPO5, YME1L1**	
	2. Cell Signaling, Nutritional Disease, Psychological Disorders (Score = 31)	**BAI1, CELSR2, DRD5, FFAR2, FZD3, Gpcr, GPER, GPR1, GPR18, GPR22, GPR37L1, GPR75, GPR101, GPR125, GPR137, GPR144, GPR146, GPR172A, GPRC5B, GRM6, MC4R, MCHR1, MTNR1A, NPBWR2, OPN1LW, OPN1SW, P2RY13, QRFPR, RXFP1, RXFP3,SCTR, SSTR5, TAAR5, TAS1R2, UTS2R**	
	3. Cellular Assembly and Organization, DNA Replication, Recombination, & Repair, RNA Post-Transcriptional Modification (Score = 31)	**ABR, ACP6, AP2S1, BANF1, C2ORF18, CD2BP2, CDK5RAP3, DDX21, DHCR7, DHX36, EWSR1, GMCL1, HMX3, IFT52, IFT57, IREB2,IFT8B, KHK, KIFAP3, LEMD3, LMNA, PREX1, PRPF8, Rac, RANBP3, RPL35, ROR1, SDHB, SEPHS1, SMNDC1, SNRPA1, SRPRB, STAMBPL1, SUN2, TOR1A**	
	4. Cell Cycle, CellularAssembly and Organization,DNA Replication,Recombination, &Repair (Score = 31)	**AIFM1, AKTIP, APEX2, AURKB, BRD4, C7ORF25, CDCA8, CENPN, CYTSB, DLG5, EEF1B2, ESPL1, FRA10AC1, GNB2, HOOK2, IMM17A, INCENP, KIF20A, NCAPG2, NPBP1, PDS5B, P-TEFb, RAD21, RCN1, RIBC2, SMC2, STAG2, STAG3, TCP11L1, TIMM10, TIMM50, TOMM34, TOMM70A, TRIM37, VDAC2**	
	5. Post-Translational Modification, LipidMetabolism, Nucleic AcidMetabolism(Score = 29)	**ACAP1, Akt, CRTAP, DHX8, DLAT, DLD, DUB, NAF1, PAIP1, PDHA1, PDK1, PDK2, PDK3, PDK4, PDP1, RBM15, SGK2, SIRT6, SNX27, SURF2, THEM4, UBXN1, USP1, USP12, USP24, USP30, USP37, USP38, USP40, USP46, USP48, UXS1, WDR20, WDR48, ZBTB2,**	

**Table 4:** IPA top canonical pathways generated from gene lists comprised of significant genes unique to day 14 in each treatment.

Significant Gene List	Top Canonical Pathways	Pathway Genes
1.0 µg/L EE2 genes uniqueto Day 14	1. Leukocyte Extravasation Signaling (ratio = 34/199; pval = 2.7E-050)	**ACTN, CRK, GNAI, F-Actin, Fer, ITGA4, ITGB1, JAM2, JAM3, JNK, MEKK4, MMP, MMP9, NCF2, NCF4, NOX, PI3K, PKC, Rac1, RAPL, RhoGAP, SDF-1, TIMP, VASP, Vav**
	2. VDR/RXR Activation (ratio = 18/81; pval = 6.1E-05)	**CEBPB, CKII, Cyclin C, CYP24A1, GADD45A, HES1, HOXA10, NCOR2, PDGFα, PKC, PPARD, Runx2, RXR, SERPINB1, WT1**
	3. Production of Nitric Oxide & Reactive Oxygen Species in Macrophages (ratio = 28/189;pval = 1.8E-04)	**AKT, CAT, GP91, HoxA10, IκB, JAK, MEKK, P40phox, P67phox, PI3K, PKC, PKCβ, PP1, PP2A, PU.1, Rac1, Rho**
	4. RAR Activation (ratio = 29/183; pval = 1.9E-04)	**AC, AKT, CDK7, CK2, COUP-TF, CRABP2, CSK, JNK, NIX1, PKA, PKC, PI3K p110, PNRC, Rac1, RALDH, RBP, RDH, RXR, SMAD, SMRT, TFIIH, TGF-β, TRUP, Vinexin**
	5. Reelin Signaling in Neurons (ratio = 17/82;pval = 2E-04)	**ApoE, CRKL, Fe65, Integrinα3β1, JNK, LIS1, NudeL, PI3K, RhoGEF**
10.0 µg/L EE2 genes uniqueto Day 14	1. Regulation of eIF4 & p70S6K Signaling (ratio = 35/135; pval = 1.6E-05)	**AKT, c-Raf, eIF3, eIF2, eIF3, eIF4A, eIF4γ, eIF4E, eIF4EBP, Integrin, MNK1, PAIP1, PI3K, PP2A, Ras, RPS6, SOS**
	2. ERK/MAPK Signaling (ratio-55/204; pval = 3.5E-05)	**14-3-3 (β, γ, η,θ, ζ), 4E-BP1, AFT-1, C3G, c-Myc, cPLA2, c-Raf, CREB, CRK, eIF4E, Elk-1, EPAC, ER, Ets, FAK, Integrin, KSR, MNK1/2, N-Myc, PKA, PP1, PP2A, PKC (α, β, γ, δ, ε, ι), SOS, STAT1/3, Talkin, VRK2**
	3. Protein Ubiquitination Pathway (ratio = 72/204; pval = 3.9E-05)	**DUB, E2, E3 cofactor, E3 HECT, E3 RING, E4, HSP, MHC class I, PSMA2, PSMA6, PSMB7, PSMB10, PSME1, PSMID, TAP**
	4. EIF2 Signaling (ratio = 31/104; pval = 5.3E-05)	**A121, AKT, c-Raf, eIF3, eIF4A, eIF4C, eIF4E, eIF4γ, HRI, PI3K, PKR, PP1c, Ras, SOS**
	5. Production of Nitric Oxide & Reactive Oxygen Species in Macrophages(ratio = 47/189;pval = 2.15E-04)	**AKT, ARG2, CAT, CBP, gp91, HoxA10, IκB, JAK, JKK, MEKK, p40phox, p67phox, PI3K, PKC, PKCβ, PP1, PP2a, PU.1, Rac1, Rho, STAT1, TNFR**

Only significant genes found in the pathway are listed.

A comparison of the molecules and genes mapped to the top 5 IPA molecular networks generated from differentially expressed genes unique on day 14 in the 1.0 µg/L and 10.0 µg/L EE2 treatments additionally indicate multiple network molecules identified in both treatments. Gene ontology of these genes indicates involvement in glycolysis, transcription and angiogenesis, translation, and ubiquitination (Table 3). It is important to bear in mind that while not all of the network genes are differentially expressed, the generated networks show relationships and connectivity between the genes suggesting involvement and possible perturbation of these signaling processes.

## Discussion

The aim of the present investigations was to achieve an integrated understanding of the effects of a 14 day exposure of EE2 on male reproductive parameters and to relate these to alterations in testicular structure and gene expression. Our data indicate 1) fertility decreased in a concentration dependent manner upon EE2 exposure, 2) morphologic alterations were time and concentration dependent and included altered spermatogenesis and thickening of the interstitium, and 3) gene expression changes were time and concentration dependent with IPA networks indicating involvement in multiple molecular pathways and signal transduction cascades.

Our reproductive assessment was designed to allow us to specifically assess male reproductive capacity without the confounding factor of exposed females. Fertility of adult males decreased in a concentration dependent manner with fertilization rates of 62.8% and 28.8%, after exposure to 1.0 µg/L and 10.0 µg/L EE2, respectively. Other studies in medaka, zebrafish and fathead minnow that assessed effects of estradiol exposure on reproduction also showed a decrease in male fertility [10], [23], [24], [38]–[44]. Interestingly, we also observed a decrease in the number of eggs laid per day following reintroduction of males post-exposure to their unexposed female breeding group in both treatment groups. The exact cause of this phenomenon is unknown, however, a previous study observed altered male courtship behavior and decreased copulation of EE2 exposed male medaka although no changes in female egg production was observed [10].

With the ability of EDCs to disrupt reproduction it is important to understand the potential ecologic implications. The reproductive impairments demonstrated in this study highlight the potential ecological impact of estrogen exposure at the population level. Whole lake exposure studies assessing fish populations in Canada to environmentally relevant EE2 concentrations found ecotoxicological effects that led to a population collapse in multiple fish species due to altered reproduction and subsequent lack of young of the year needed to maintain the populations [19], [20]. Our study adds further weight to the male role in impaired reproduction by demonstrating that estrogen exposure compounded reproductive effects through decreased fertilization rates and fecundity in females, possibly due to behavioral alterations in males.

The histologic analysis of testes from the exposed non-breeding males found a decrease in early stage germ cells (spermatogonia and spermatocytes), an increase in apoptotic germ cells and a generalized thickening of the interstitium. Previous studies report similar findings to ours regarding altered testicular histology following estradiol exposure in fish, including fathead minnow, zebrafish and medaka [10], [24], [40], [42], [44], [45]. While the exposure time and concentrations vary, the histological alterations observed are similar to the changes reported here including altered spermatogenesis, testicular disorganization, testicular oocytes, thickening of the interstitium and fibrosis.

Our histologic assessment of the testis following the breeding experiment indicate similar alterations. However, these actively breeding males had an accentuated testicular morphology compared to their EE2 exposed, nonbreeding counterparts. Even the control breeding males had changes in the gonad including an increase in early stage germ cells and a slight thickening of the interstitium. This is likely due to an overall decreased volume of mature sperm indicating active reproduction. A histologic comparison of the EE2 treated breeding and non-breeding males indicates that the EE2 treated breeding males had a furthered morphologic change compared to their non-breeding counterparts (males depurated for 14 days in the absence of females). It is important to keep in mind that breeding males actively expelled mature sperm, evident by successful fertilization, albeit decreased in the EE2 treated breeding males. Continuous spermatogenesis is needed to replenish spermatozoa reserves that fill the efferent duct space. We hypothesize that the active dispelling of spermatozoa from the breeding males plays a role in the appearance of accentuated thickened interstitium observed in both control and treated animals due to a decrease of late stage germ cells occupying lobule and efferent duct space. In the 1.0 µg/L treatment group, the non-breeding males allowed to depurate unaccompanied by females showed signs of recovery while males in the presence of females had the most significant testicular changes of this treatment group, including a further increase in interstitial thickening and a decrease in germinal epithelium. In the 10.0 µg/L treatment group, following the depuration period there was no sign of recovery in the non-breeding males. However, breeding males had even further morphologic degeneration with, again, severe thickening of the interstitium, and significant depletion of germinal epithelium accompanied by altered spermatogenesis.

We also sought to link EE2 induced altered testicular morphology to possible perturbation of proper cell signaling due to altered gene expression. The IPA networks allowed us to identify functional testicular gene networks and signaling pathways associated with the EE2 exposure. While no histologic changes were observed in the testis on day 1 of exposure, gene expression changes involving critical functions were observed. This indicates that EE2 induced gene expression changes precede morphologic changes in the testis. This same pattern of gene expression changes preceding altered morphology was previously described in the liver of medaka following TCDD exposure [46]. The pattern of change is observed with genes involved in apoptosis and cell proliferation and subsequent altered spermatogenesis as well as in angiogenesis, wound healing and collagen production and subsequent thickening of the interstitium.

When comparing IPA networks for genes common to all sampling days in the 1.0 µg/L and 10.0 µg/L EE2 treatment groups, multiple genes and molecules involved in hormone signaling and male reproduction were mapped to the networks including CYP19a (aromatase), FSH (follicle stimulating hormone), Insulin, LH (luteinizing hormone), NR0B1 (DAX1), NR0B2 (SHP), NR5A1 (SF1), and SOX9. All are integral to the endocrine system, proper Leydig and Sertoli cell function and subsequently reproduction (for reviews see [31], [47]–[53]. With the many feedback mechanisms involved with hormone homeostasis and subsequent male reproductive function, it is anticipated that hormone signaling would be altered through the entirety of the EE2 exposure.

Testicular function is dependent on proper hormone signaling and a tightly controlled balance between cell proliferation and apoptosis. Our observations of decreased germ cells and increased apoptotic cells reflect changes in gene expression and signaling pathways associated with cell proliferation, cell cycle and apoptosis. The IPA networks of significant genes common to all days in the 1.0 µg/L and 10.0 µg/L treatments identified multiple genes associated with established signaling pathways important to these functions including ERK1/2 MAPK, p38 MAPK, PDGF, and TGFβ (Table 2). These genes and their associated pathways are interdependent with critical cross-talk between pathways important in coordinating cellular responses. Interestingly, KEGG pathway analysis (www.genome.jp/kegg/) indicates various combinations of these genes found together in multiple pathways but all are found within the MAPK pathway (map04010; http://www.genome.jp/kegg-bin/show_pathway?map04010). The MAPK and PI3K/Akt signaling pathways are responsive to estrogen signaling and play an integral role in maintaining the balance of cell proliferation and apoptosis in multiple cell types within the testis [54–60,61a,62–64]. This signaling has been shown to be particularly important in germ cells and Sertoli cells including cell cycle progression of spermatogonia, spermatocytes and spermatids and Sertoli-germ cell adheren junctions. These gene signaling pathways also have roles or engage in crosstalk with pathways involved in the other biological functions which were also identified in the IPA network including wound healing, inflammation and cytoskeleton organization.

**Figure 8:**
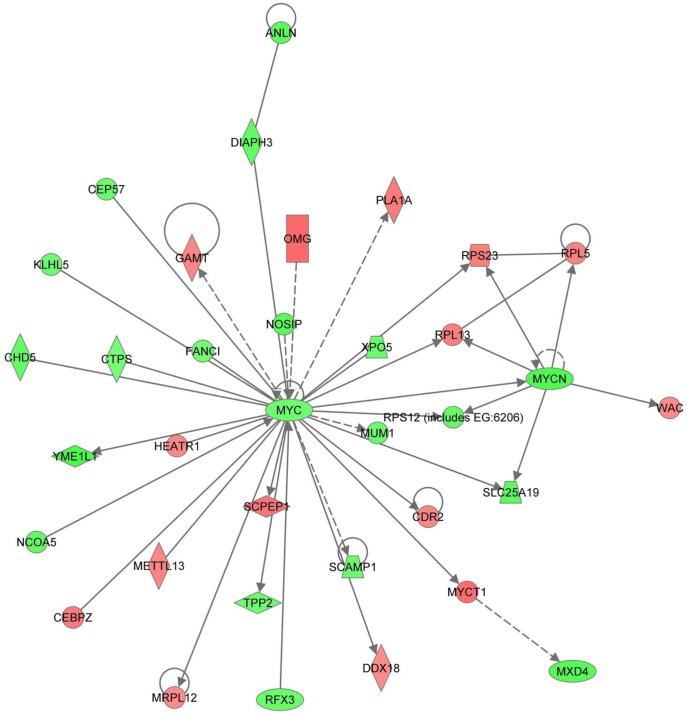
Top ranked IPA Molecular Network generated from differentially expressed genes unique to day 14 in the 10.0 µg/L EE2 treatment. Myc family of genes is central to this Molecular Network suggesting an important role in the observed changes in gene expression and histology on day 14 in the 10.0 µg/L treatment group.

ERK/MAPK signaling also appeared in the IPA assessment of significantly different genes unique to day 14 in both treatments when histologic changes are most extensive. In the 10.0 µg/L treatment group, the ERK/MAPK pathway was the second ranked canonical pathway with multiple associated transcription factors differentially expressed including ESR, ETS, STAT1/3, ELK1, c-Myc, and N-Myc. Furthermore, of particular interest is the central role of Myc family of oncogenes in the top molecular network (Figure 8). This suggests that the disruption of the Myc gene family has an important role in changes observed on day 14 in the 10.0 µg/L treatment group which has the most significant reproductive effect, testicular alterations and no evident recovery. Myc is activated with various mitogenic signals including MAPK/ERK and PI3K/AKT and broadly influences genes important to many functions involved in cell proliferation, cell growth, apoptosis, differentiation and stem cell renewal, including spermatogonial stem cell proliferation [65]–[72]. Furthermore, transcription targets of Myc or other transcription regulators with which Myc partners, were differentially expressed in our study including: TERT, BRCA1, CDKs (cyclin dependent kinases), EIF4s (eukaryotic translation initiation factors), EEFs (eukaryotic translation elongation factors), E2Fs (transcription factors), histone H4, histone deacetylases (HDACs), RPLs and RPSs (ribosomal protein large and small subunits), NCOR, NCL, APEX1, and DDX18 [67], [73]–[78]. An emphasis on Myc genes further suggests that the MAPK and PI3K/AKT pathways are important to the testicular effects seen from the EE2 treatment and may indicate the extent to which these and associated pathways are disturbed in the 10.0 µg/L EE2 treatment group.

**Figure 9:**
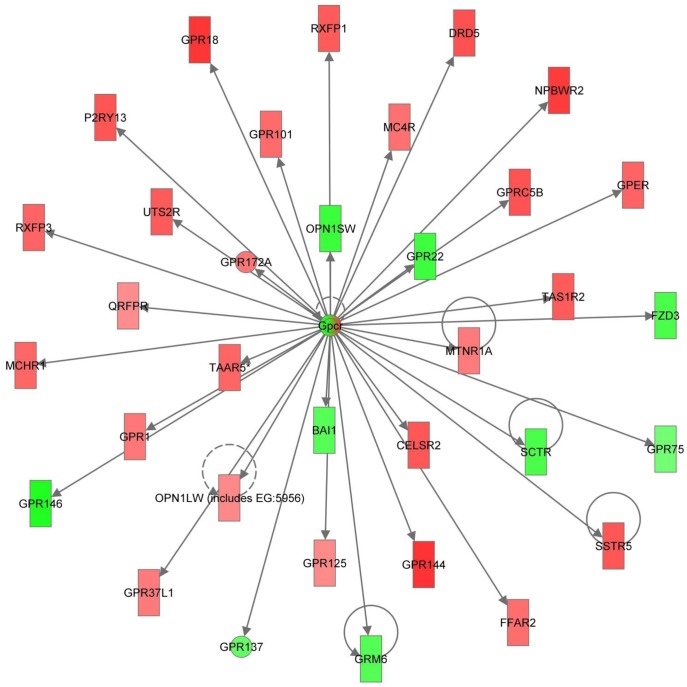
Second top ranked IPA Molecular Network generated from differentially expressed genes unique to day 14 in the 10.0 µg/L EE2 treatment. G-protein coupled receptors (GPCRs) are emphasized in this Molecular Network indicating a significant effect on G-protein signaling pathways including the G-protein estrogen receptor (GPER) on day 14 in the 10.0 µg/L treatment group.

The second top molecular network of genes unique to day 14 in the 10.0 µg/L treatment group centers on G protein-coupled receptors (GPCRs; Figure 9). Many significantly different GPCRs are part of this network, including the G protein estrogen receptor (GPER). GPCRs are a large family of proteins important in multiple signal transduction pathways whose agonists include neurotransmitters, hormones, chemokines, and bioactive lipids [79], [80]. Not surprisingly, GPCRs are important regulators of cell survival, apoptosis, movement, proliferation, differentiation and growth. Additionally, all four classes of G proteins are able to regulate MAPK and PI3K/AKT pathways and subsequent transcription factors through multiple mechanisms of action [79], [80]. Estrogen signaling occurs through both the classical (genomic) estrogen signaling pathway, as well as through nongenomic signaling in which estrogen rapidly activates protein kinases (MAPK, PI3K, and PKC), adenylate cyclase, calcium and cAMP [81], [82]. This has been shown to be regulated through membrane bound ESR and the aforementioned GPER [83], [84]. Additionally, there has been extensive data, particularly in breast cancer research, demonstrating crosstalk between estrogen signaling and ErbB2/HER2/neu, a membrane tyrosine kinase epidermal growth factor receptor, regulating MAPK and AKT signaling [85]. ErbB2 is differentially regulated on day 14 in both the 1.0 and 10.0 µg/L treatments. This suggests that perturbation of both genomic and nongenomic estrogen signaling networks in this model.

Microarray assessments of EE2 induced testicular gene expression changes carried out in zebrafish and medaka find multiple genes in common to our results [23], [27]. Hirakawa et al. [27] identified candidate genes of testicular ooctyes in EE2 exposed adult male medaka through microarray analysis. Interestingly, multiple genes which they emphasized, were also differentially expressed in our study including zona pellucida genes (ZP1, ZP3), vitellogenin 1 (VTG1), FIGalpha (FIGLA), and eukaryotic translation initiation factor 4E family member 3 (EIF4E). Real-time PCR follow up of their candidate genes suggests that Zpc5 is the most reliable testicular oocyte marker gene, which is not significantly different in our study. This is to be expected since testicular oocytes were not frequently observed during our 14 day EE2 exposure.

Similar to Santos et al. [23], our study found that ubiquitin genes were differentially regulated with time and concentration and identified in IPA molecular networks. During spermatogenesis, ubiquitin-dependent proteolysis is important for cellular condensation of germ cells [86]. Perturbation of this ubiquitin process has been implicated in the degradation of sperm quality [87]. While Santos et al. [23] did not note any histological alterations in the testis, sperm quality analysis indicated decreased motility. They suggest the EE2 induced alterations of gene expression profiles in the ubiquitin system and in glutathione peroxidase are likely mechanisms that led to lower sperm quality, and decreased fertilization success observed in the males in their study. Our data also suggests that altered ubiquitination may also play a role in the observed altered testicular function and decreased fertility.

Together, these microarray studies assessing EE2 induced gene expression changes in the testis highlight the importance of pairing the results with morphologic alterations. There is a continuum of gonadal changes that develop following EE2 exposure dependent on dose and time, as evident by results observed. Each of these described gene expression analyses, while having some similarities, have many differences. When put into context with their respective histologic assessments a more complete depiction is possible. Furthermore, this range of altered testicular morphology is reported in the literature and therefore understanding the associated range of gene expression changes, assumes additional relevance. For example, in the previously described whole lake EE2 exposure study, testicular tissue of fathead minnows collected early in the study showed similar altered morphology to our study including delayed spermatogenesis, widespread fibrosis and a decrease in spermatocytes [19]. However, in subsequent years testicular tissue had a higher prevalence of testicular oocytes. This ecological study is the quintessential example of the spectrum of EE2 induced altered testicular morphology and further illustrates the importance of a thorough understanding of the associated gene expression changes. Toxicity studies should ultimately strive for a comprehensive assessment of molecular, pathologic and apical repercussions.

The goal of this study was to link reproductive impairments and altered testicular morphology induced by EE2 exposure in male medaka to testicular gene expression changes. We observed decreased fertility in both treatment groups along with time and concentration dependent morphologic changes including thickened interstitium and altered spermatogenesis. Finally, analysis of testis specific gene expression and pathway analysis anchored the alterations in structure and function of this male reproductive organ. Pathway analysis of significantly different genes from the microarray data suggests differential expression of genes involved in angiogenesis and wound healing, apoptosis, cell adhesion, cell death, cell cycle and proliferation, collagen, cytoskeletal organization, hormone signaling, male gonad function, and ubiquitination among others. There is more to be done, especially in regard to a further detailed understanding of the altered signaling pathways and subsequent sequence of events that lead to altered testicular morphology in exposed individuals. However, the groundwork is now established for an integrative understanding of the role of EE2 on testis structure and function.

## Materials and Methods

### Ethics Statement

Experiments were conducted using approved Duke University Institutional Animal Care and Use Committee protocols.

### Chemicals

EE_2_ (98% purity, Fluka, St. Louis, MO) was used to prepare nominal stocks (0.004, 0.04, and 0.4 mg/ml) in dimethyl sulfoxide (DMSO). The stocks were stored at room temperature in the dark.

### Medaka

Orange-red (OR) outbred-medaka fish (*Oryzias latipes*) were maintained at the Duke University Aquatic Research Facility under standard recirculating water conditions following approved animal care and maintenance protocols (Duke University Institutional Animal Care and Use Committee). Water temperature and pH were monitored daily and maintained at ∼25°C and ∼7.4, respectively, and broodstock were under a strict light:dark cycle of 16∶8 hours. Dry food (Otohime B1, Reed Mariculture, Campbell, CA) was fed several times per day via automated feeders with once daily supplementation of newly-hatched *Artemia nauplii*. Adults reared under the above conditions were used in all aspects of this study including gene expression, histology and reproduction (see below).

### Reproductive Assessment

This consisted of three time periods: pre-exposure, male exposure, and post-exposure. Throughout all three time-periods, egg production and fertilization rate were monitored.

#### Pre-exposure

Reproductively active, 5–6 month old adult medaka were placed randomly in breeding groups consisting of three females and one male (25 breeding groups total). Each breeding group was maintained in a 2-L glass beaker with 1800 mL of embryo rearing medium (ERM; [88]. Daily renewal of 75% of the medium was performed. Each breeding group received approximately 2 mg/day dry food (Otohime, Reed Mariculture, Campbell, CA, USA) and artemia nauplii twice per day. Beakers were arranged randomly in isolation and maintained under a 16∶8 light:dark cycle for 12 days. Eggs were collected and fecundity of each breeding group was established daily. Fertilization rate of each group was determined under a dissecting microscope according to procedures previously described [89], [90]. After the pre-exposure period, each breeding group was randomly assigned to one of three treatments groups.

#### Male exposure

Each male was removed and placed individually in a designated 500-mL beaker for the male exposure period. Males were treated with 500 mL of spiked ERM with either DMSO (control)0op, 1.0 µg/L EE2 or 10.0 µg/L EE2 for 14 days with a 50% renewal every 48 hours. Males were fed approximately 0.5 mg/day dry food and artemia nauplii daily and maintained under a 16∶8 light:dark cycle. Throughout the male exposure, females were maintained in 2-L glass beakers with 1800 mL ERM as described in the pre-exposure period. Fecundity and fertilization rates of females were monitored during this time.

#### Post-exposure

Individual males were returned to their original beaker for breeding to maintain consistency of breeding groups. For each breeding group, fecundity and fertilization rate were recorded on days 1–14, 17 and 20 of the post exposure period. Water conditions were maintained in the same manner as above, including the daily 75% water renewal. This design made it possible to relate morphology of testis to each individual’s fertilization success prior to and following exposure. On day 20 post-exposure of the breeding experiment, males were anesthetized in ice-cold ERM and the testis was removed and processed for paraffin based histological analysis as described below.

### Statistics

One-way analysis of variance, followed by Tukey HSD post-hoc test was performed to assess treatment effects of EE2 exposures using JMP 8 (SAS Institute Inc.). The number of eggs produced/day, number of fertilized eggs/day and the fertilization percentage of eggs laid/day were used to analyze differences between treatment groups. All data in figures are presented as the mean ± SEM. *P*<0.05 was considered significant.

### Histological Analysis and Gene Expression

#### Exposures

Treatment exposures were completed in triplicate including DMSO (vehicle control), 1.0 µg/L EE2, and 10.0 µg/L EE2. Fish were sampled for histology and gene expression on days 1, 7 and 14 of exposure and for histology only on day 14 post-exposure depuration (recovery). For histologic analysis, 2-liter beaker replicates received 23 individuals each for exposures for the days 1, 7 and 14 time point and a separate set of beakers received 11 fish for the post-exposure day 14 depuration time point. For gene expression analysis, 5 male fish were placed in 2-liter beaker replicates for each treatment and sampling time point. ERM was spiked with the appropriate EE2 stock (0.0025% of total volume) and equally distributed between the 2-L beakers for a total of 2 L spiked ERM per beaker with a 50% renewal of spiked ERM every other day for 14 days. Following the 14 day exposure, fish for histologic analysis were placed into fresh ERM for 2 days and then moved into a flow-through system until time of sacrifice, i.e. 14 days post-exposure. The fish were maintained under a 16∶8 hour light:dark cycle and fed ad libitum the dry diet as above on alternate days.

#### Histological analysis

This was performed as follows: high resolution on control animals for general testicular anatomy and morphology; analysis of males on day 1, 7 and 14 of EE2 exposure and day 14 post-exposure depuration (recovery); and analysis of actively breeding medaka following EE2 exposure as described above in the reproductive assessment.

#### High resolution methods

Adult male 6 month old fish collected from our colony were anesthetized in ice-cold ERM. The testis was removed and fixed in a cocktail of 0.5% glutaraldehyde, 2% paraformaldehyde, 1% sucrose and 1% CaCl_2_ in Histochoice (Amresco, Solon, Ohio) for 24 hours at 4°C followed by immersion in Holt’s gum sucrose solution (1% aqueous gum arabic and 30% aqueous sucrose) at 4°C for ≥24 hours [91]. Tissue samples were then embedded in glycol methacrylate, sectioned at 2.5 µm thickness, mounted on glass histological slides and stained with hematoxylin and eosin.

#### Paraffin embedment

At each sampling time-point during exposure, 1–2 fish from each replicate beaker were anesthetized in ice-cold ERM, and the testis was collected for histology. Testes were fixed in 2% paraformaldehyde/phosphate buffered saline (PBS) for a minimum of 72 hr at 4°C and stored in 6% sucrose/PBS at 4°C until time of processing. Following dehydration in a graded series of ethanol (70% (×1), 85% (×1), 95% (×1) and 100% (×3) for 10 minutes each) and clearing in xylene (10 min ×3) tissues were embedded in paraffin (Paraplast® Plus, McCormick Scientific, St. Louis, MO). 5-µm thick, longitudinal serial sections were cut using a Microm HM 355 S microtome (Thermo Fisher Scientific, Walldorf, Germany) through the entire organ, mounted on histological slides and stained with hematoxylin and eosin for analysis.

### RNA Isolation

For gene expression, the testis of three fish per replicate beaker (n = 3) were removed as previously described [46], [92], pooled and immediately frozen in liquid nitrogen for RNA isolation. In short, three pooled testes were homogenized with 1 ml RNA Bee (TelTest, Friendswood, Texas) using a Polytron homogenizer (Kinematica, Bohemia, New York) cleaned with RNaseZAP (Sigma, St. Louis, Missouri), diethylpyrocarbonate (DEPC) treated water, and sterile de-ionized water. Total RNA was purified from the homogenate using RNeasy Mini Kit (Qiagen, Valencia, California) followed by an on-column-digest with DNase to eliminate DNA contamination using RNase-free DNase Set according to manufacturer’s instructions (Qiagen, Valencia, California). The sample was then eluted with 30 µl RNase-free water (52°C). Total RNA samples were stored at −80°C. RNA concentrations were assessed using the Agilent 2100 Bioanalyzer (Agilent Technologies, Santa Clara, California). Each sample was analyzed for gene expression using one medaka array per biological sample (n = 3).

### Array Production

Probes were produced by mining the medaka Ensembl Genome Browser using the biomart function for all annotated medaka genes based on the MEDAKA1 (October 2005) assembly provided by the National Institute of Genetics (NIG) and the University of Tokyo. This resulted in 15,207 predicted gene sequences. All medaka genes were assigned homologs to the Human Ensembl Genebuild 36 (http://useast.ensembl.org/Homo_sapiens/Info/Index?db=core). Seventy-mer oligo probes were designed using the eArray portal with defined quality control parameters for both cross hybridization and base composition score. RNA was amplified using the Low RNA Input Fluorescent Linear Amplification Kit (Agilent); and was annealed with a primer containing a polydT and a T7 polymerase promoter for reverse transcription and first and second strand cDNA synthesis. cRNA was produced using T7 RNA polymerase and incorporated cyanine-5 (Cy5) labeled CTP. The quality of the labeled cRNA was verified and concentration was measured spectrophotometrically. Control or experimental cRNA (0.75 µg) was hybridized to each array as a single channel hybridization. Hybridization was conducted on a custom 15K ×8 Agilent medaka array using the “*In situ* Hybridization Kit-Plus” (Agilent) at 60°C for 17 h. The arrays were washed according to Agilent's SSPE wash protocol using a solution of 6× SSPE, 0.005% *N*-lauroylsarcosine, followed by a solution of 0.06× SSPE, 0.005% *N*-lauroylsarcosine, and Agilent's Stabilization and Drying Solution. The arrays were scanned on an Agilent G2565BA Microarray Scanner and data from the scans were compiled with Agilent Feature Extraction Software 8.1.

### Microarray Statistical and Pathway Analysis

Analysis of the microarray data was performed using JMP Genomics 4.1(SAS Institute Inc, Cary, North Carolina). Data was log2 transformed during the import process and normalized using the standard normalization routine implemented in JMP Genomics 4.1. A distribution analysis was conducted for quality control purposes prior- and subsequent to normalization and alignment of the overlay plots was used as an indicator of high quality data. Data analysis was performed by conducting a principal component analysis (PCA) by day and treatment using time-matched treatment-to-control differences calculated from standard least-square mean. An ANOVA was performed to test for statistical differences between treatment and control groups on a day by day basis. The False Discovery Rate (FDR) at alpha 0.05 was used to account for the multiple testing problem. Hierarchical clustering was performed using the significant gene sets derived from the ANOVA analysis data set. Microarray gene expression data was deposited in Gene Expression Ominbus (GEO; http://www.ncbi.nlm.nih.gov/geo).

Genes for which differential expression was significant were further analyzed through the use of Ingenuity Pathway Analysis (IPA; Ingenuity® Systems, www.ingenuity.com). A data set containing significantly different genes based on our ANOVA analysis with their corresponding gene identifiers was uploaded into the application and used for molecular network and canonical pathway generation. Each identifier was mapped to its corresponding object in Ingenuity’s Knowledge Base and molecular networks were generated based on their connectivity. Canonical pathways most significant to the data set were identified, from Ingenuity Pathways Analysis library. The significance of the association between the data set and the canonical pathway was measured in 2 ways: 1) A ratio of the number of molecules from the data set that map to the pathway divided by the total number of molecules that map to the canonical pathway; and 2) Fisher’s exact test, to calculate a p-value determining the probability that the association between the genes in the dataset and the canonical pathway is explained by chance alone.
